# Post-stroke Cognition at 1 and 3 Years Is Influenced by the Location of White Matter Hyperintensities in Patients With Lacunar Stroke

**DOI:** 10.3389/fneur.2021.634460

**Published:** 2021-03-01

**Authors:** Maria del C. Valdés Hernández, Tara Grimsley-Moore, Francesca M. Chappell, Michael J. Thrippleton, Paul A. Armitage, Eleni Sakka, Stephen Makin, Joanna M. Wardlaw

**Affiliations:** ^1^Centre for Clinical Brain Sciences, UK Dementia Research Institute at the University of Edinburgh, Edinburgh, United Kingdom; ^2^College of Medicine and Veterinary Medicine, University of Edinburgh, Edinburgh, United Kingdom; ^3^Academic Unit of Radiology, University of Sheffield, Sheffield, United Kingdom; ^4^Centre for Rural Health, University of Aberdeen, Aberdeen, United Kingdom

**Keywords:** recent small subcortical infarct, lacunar, stroke, white matter hyperintensities, cognition

## Abstract

Lacunar strokes are a common type of ischemic stroke. They are known to have long-term cognitive deficits, but the influencing factors are still largely unknown. We investigated if the location of the index lacunar stroke or regional WMH and their change at 1 year could predict the cognitive performance at 1 and 3 years post-stroke in lacunar stroke patients. We used lacunar lesion location and WMH-segmented data from 118 patients, mean age 64.9 who had a brain MRI scan soon after presenting with symptoms, of which 88 had a repeated scan 12 months later. Premorbid intelligence (National Adult Reading Test) and current intelligence [Addenbrooke's Cognitive Exam-Revised (ACE-R)] were measured at 1, 12, and 36 months after the stroke. ANCOVA analyses adjusting for baseline cognition/premorbid intelligence, vascular risk factors, age, sex and total baseline WMH volume found that the recent small subcortical infarcts (RSSI) in the internal/external capsule/lentiform nucleus and centrum semiovale did not predict cognitive scores at 12 and 36 months. However, RSSI location moderated voxel-based associations of WMH change from baseline to 1 year with cognitive scores at 1 and 3 years. WMH increase in the external capsule, intersection between the anterior limb of the internal and external capsules, and optical radiation, was associated with worsening of ACE-R scores 1 and 3 years post-stroke after accounting for the location of the index infarct, age and baseline cognition.

## Introduction

Lacunar strokes make up 20–30% of all ischemic strokes (1). Despite smaller in size than most cortical strokes, they have been associated with long-term disability, physical (2), gait, and balance impairments (3), and progressive motor deficits, these defined as the deterioration of National Institutes of Health Stroke Scale (NIHSS) motor score ≥ 1 during the first 7 days after admission (4). After a lacunar stroke, there is also often a deterioration in cognitive function and memory (5), increased occurrence of depression (6), reduced spatial awareness (7, 8), and dementia progression (9). However, the impact of lacunar infarct on cognition alone is still unclear. Two studies reported no association between lacunar infarcts and subjective memory complaints (10, 11) while another reported an association specifically with infarcts located in the basal ganglia (12), thus suggesting that lacunar infarct location is important in post-stroke cognition.

The general impact of infarct location on cognition has been studied in relation to stroke lesion volume (13), shape (14), white matter hyperintensity (WMH) volume (15), post-stroke depression and behavioral disorders (16). A large multi-center study found that infarcts (i.e., cortical and subcortical) affecting the basal ganglia and internal capsule were associated with impairment of global cognition (17), in a sample of 878 patients with ischemic stroke. Ischemic strokes' lesions in both brain hemispheres have been associated with lower performances in visuospatial and executive functions (7). Left hemisphere stroke lesions have been associated with higher incidences of depression (18); and behavioral changes post-stroke have been correlated to changes within specific brain regions (16, 19). However, spatial associations of ischemic lesions with cognition vary with time. A study on 76 stroke patients reported a spatial pattern of acute ischemic lesion clusters associated with cognitive outcome at 1–3 months post-stroke being no longer associated with outcome at 1 year (15).

Specifically the effect of lacunar infarct location in post-stroke cognition remains poorly understood. Added to confounding factors in the classification (20), manifestation and recognition (21) of lacunar strokes, variations in their evolution [i.e., cavitation, disappearance, progression to a WMH (22)] limit attempts to understand their impact on cognition. Lacunar stroke lesions are a manifestation of small vessel disease (SVD), and present together with other neuroradiological features like enlarged perivascular spaces, brain microbleeds and WMH. The latter is thought to be a main indicator of SVD severity. While the effect of WMH volume at the time of stroke is a well-established predictor of cognition post-stroke (23–25), the impact of the location of WMH combined with that of the acute stroke lesion, or of WMH change after the stroke, on the post-stroke cognitive outcome of patients with lacunar stroke is still unknown. After a minor stroke some WMH increase while some regress (26). Improvements in the prediction of cognitive and brain ischemic changes after a lacunar stroke could help to target future treatment.

The aims of this study are to: (1) evaluate the influence of lacunar stroke lesion location on cognition over a 3 year period after the lacunar stroke; and (2) explore the effect of WMH spatial distribution and change in the year after stroke on cognition, accounting for the location of the acute lacunar infarct. Given that symptomatic recent small subcortical infarcts (RSSI) are mainly located in the vicinities of the corticospinal tracts (27), related with motor and sensory functions, we hypothesized that RSSI location alone would not be associated with cognition. However, we hypothesize that stroke lesion location will differentially influence the association between the spatial distribution of WMH change at 1 year and post-stroke cognition, accounting for vascular risk factors and age at the time of stroke. In other words, we hypothesize that RSSI location will have an indirect effect in post-stroke cognition in patients with lacunar stroke.

## Materials and Methods

### Subjects

We analyzed imaging, cognitive and clinical data from all patients presenting prospectively to a regional hospital with lacunar stroke syndrome (*n* = 118, 67 males, 51 females) during the years 2010 until 2013, who participated in a longitudinal study of mechanisms of lacunar stroke (28). Patients were excluded if they lacked the capacity to consent, had a medical condition that made follow-up clinical assessment unlikely or impossible, magnetic resonance imaging (MRI) baseline examination did not confirm the stroke diagnosis, or if had severe renal impairments. This analysis further excludes patients that presented with a cortical (i.e., non-lacunar) acute stroke. Protocols for the primary study were approved by the Lothian Ethics of Medical Research Committee (REC 09/81101/54) and NHS Lothian R+D Office (2009/W/NEU/14), on the 29th of October 2009 (28, 29).

### Vascular Risk Factors

Our analyses considered medically-diagnosed hypertension (yes, no), hyperlipidemia (yes, no), and smoker status (current, recent i.e., <1 year, non-smoker, ex-smoker i.e., more than 1 year) and diabetic status (yes, no), collected at diagnosis.

### MRI Acquisition

All MRI scans were acquired in a 1.5T GE Signa Horizon HDxt clinical scanner (General Electric, Milwaukee, WI, USA) operating in research mode and using a self-shielding gradient set with maximum gradient of 33 mT/m and an 8-channel phased-array head coil (30). We use data derived from analyzing diffusion weighted imaging (DWI), fluid attenuation inversion recovery (FLAIR), T2-, T1-, and T2^*^-weighted MRI, acquired with identical protocols soon after presenting to hospital with acute stroke symptoms and 1 year after (29). Imaging data were not acquired at the 3 year time-point.

### Image Analysis

All image analysis was performed blinded to clinical, cognitive and other outcome data. All segmentations were done in a common image space patient-specific, to which baseline and 1 year image sequences were co-registered using the linear registration tool from the FMRIB Software Library (FSL-FLIRT) (31).

#### Stroke Subtype and Lesion Location

All MRI were assessed by an experienced neuroradiologist who coded the location, type, distribution and size of all infarcts, new and old, and their change on MRI at 1 year (i.e., if cavitated, disappeared, increased, or decreased in size) (22, 30). We only used the type and location of the recent and old subcortical infarcts in our analyses, coded as per Wardlaw and Sellar (32) and The IST-3 Collaborative Group (33) by an experienced neuroradiologist (JMW) (proforma available from www.ed.ac.uk/edinburgh-imaging/analysis-tools/stroke). The anatomical locations were: internal and external capsule/lentiform nucleus, internal border zone, centrum semiovale, thalamus, brainstem, cerebellum, and optical radiation.

#### WMH and Stroke Lesion Volumes

We segmented the WMH and stroke lesions at baseline and follow-up following the protocol from Valdes Hernandez et al. (30). Briefly, stroke lesions (old and new), were segmented semi-automatically on FLAIR, using thresholding combined with a region-growing algorithm (34) in Analyze^TM^ 11.0 (https://analyzedirect.com/). WMH and other brain tissues were segmented using a multispectral approach (30). Volumes were recorded at baseline and 1 year follow up. For statistical analysis, volumes, obtained from the binary masks, were calculated as a percentage in the intracranial volume (% ICV). This was also segmented semi-automatically on co-registered baseline T2^*^-weighted images using also Analyze^TM^ 11.0 software.

#### Voxel-Based Analysis

All T1-weighted images were semi-rigidly mapped in the standard space (35) using non-linear registration (http://sourceforge.net/projects/niftyreg/) through TractoR (http://www.tractor-mri.org.uk/diffusion-processing), and the transformation matrix was applied to the WMH binary masks. We generated a map of WMH change from baseline to 1 year for each patient coding the voxels in three classes as those with new WMH at 1 year, those with WMH at both time points, and those in which WMH disappeared at 1 year. We, then, generated a 4D array after concatenating the WMH change maps from all patients for statistical analysis.

### Cognitive Assessment

We used the total scores of the Addenbrooke's Cognitive Examination-Revised (ACE-R) and the National Adult Reading Test (NART) applied 1 to 3 months after the initial stroke (baseline), 1 year later (1 year follow-up) and 3 years after the initial stroke (3 year follow-up) (36, 37).

### Statistical Analysis

Using MATLAB R2019b and Statistical Package for Social Science (SPSS version 25), we performed analysis of frequencies of RSSI and old small subcortical infarcts per anatomical region. We compared age, baseline imaging and vascular risk factors of patients who provided cognitive data at each time point against those who did not provide these data using the Mann-Whitney U test (i.e., non-parametric test of two independent non-paired groups).

We used analysis of covariance (ANCOVA) to explore the effect of the RSSI location in cognitive scores at 1 and 3 years follow-up examinations. In the models, post-stroke cognition was the outcome variable, and baseline cognition and presence/absence of RSSI at specific locations were the predictors. Models were adjusted for age, baseline WMH volume, premorbid cognition (NART) (38), and vascular risk factors (diabetes, hypertension, hyperlipidemia and smoker status). Given reduction in sample size at follow-up examinations bootstrapping was performed.

We implemented voxel-wise regression models to explore whether WMH change influenced post-stroke cognition at 1 and 3 years, accounting for the acute stroke lesion (RSSI) location, coded to include location of primary and/or secondary DWI-positive clusters, using a machine-learning approach. We used the MATLAB function “fitrlinear” to fit a regularized support vector machine regression model with a ridge penalty type optimized through a stochastic gradient descent approach for accuracy. This approach was selected due to the high-dimensionality and sparsity of the predictor data. The code is available and documented in (39). The outcome variable was the ACE-R scores at 1 or 3 years post-stroke. Covariates were age, premorbid cognition (i.e., baseline NART scores) and the RSSI location code. Analyses were repeated (1) adding vascular risk factors, (2) not considering the RSSI location but instead the WMH volume at baseline, (3) considering RSSI location, baseline WMH volume and number of lacunes, and (4) considering, in addition, the volume of old stroke lesions, as covariates in the models. The regularization term strength was set at 1/47.

## Results

### Sample Characteristics

#### Demographics

From the 118 patients that had an MRI at baseline, 68 provided cognitive data. From the 90 that had an MRI at 1 year follow-up, 88 had valid MRI-derived data (two MRI scans were incomplete) and 63 provided cognitive data. Sixty six patients provided cognitive data 3 years after the stroke (Table 1). Reasons for absence of data are provided in (40) and McHutchison et al. (36). The mean age of the sample at baseline was 64.93 (SD 11.75, 95% CI [62.76 67.091]) years old and did not differ from the age of subsamples tested at 1 and 3 years (64.79 (10.87 [62.47 67.10]) years old and 64.25 (10.45 [61.68 66.83]) years old, respectively) (Table 1). The proportion of males to females in all three recordings was similar with marginally more males in the two first visits.

**Table 1:** Sample characteristics.

**Variable type**	**Baseline measurements**			**1 year follow-up measurements (*n* = 88)**	**3 year follow-up measurements (*n* = 66)**
	**Baseline sample (*n* = 118)**	**1 year follow-up subsample (*n* = 88)**	**3 year follow-up subsample (*n* = 66)**		
**Age (years) [mean(SD)]**	64.93 (11.75)	64.79 (10.87)	64.25 (10.45)		
**Gender [% (*****n*****)]**					
Male	67 (57)	52 (59)	33 (50)	52 (59)	33 (50)
Female	51 (43)	36 (41)	33 (50)	36 (41)	33 (50)
**Brain measurements**					
ICV (ml) [mean (SD)]	1469.20 (139.82)	1469.04 (140.87)			
Old stroke lesion volume (ml) [median (IQR)]	1.25 (0.53–3.17) (*n* = 42)	1.29 (0.65–4.18) (*n* = 29)	1.25 (0.48–4.35) (*n* = 20)	1.51 (0.71–3.84) (*n* = 26)	
Index stroke lesion volume (ml) [median (IQR)]	1.17 (0.70–1.63) (*n* = 77)	1.17 (0.73–1.78) (*n* = 61)	1.22 (0.72–1.78) (*n* = 41)	0.68 (0.35–1.27) (*n* = 56)	
Total WMH volume (ml) [median (IQR)]	14.37 (4.55–36.13) (*n* = 117)	14.56 (5.55–34.30)	11.35 (4.0–24.52)^*^ (*n* = 65)	16.51 (7.12–35.44)	
**Clinical history (vascular risk factors) at baseline [*****n*** **(%)]**					
Diabetes	12 (10.2)	8 (9.0)^*^	3 (4.5)^*^		
Hypertension	82 (69.5)	65 (73.9)	48 (72.7)		
Hyperlipidemia	73 (61.9)	56 (63.6)	40 (60.6)		
Current smoker	46 (39.0)	33 (37.5)	21 (31.8)		
Recent smoker	5 (4.2)	3 (3.4)	1 (1.5)		
Ex-smoker	31 (26.3)	22 (25.0)	19 (28.8)		
Non-smoker	35 (29.7)	30 (34.1)	24 (36.4)		
**RSSI location [*****n*** **(%)]**					
Internal/external capsule lentiform nucleus	21 (17.8)	16 (18.2)	13 (19.7)		
Internal border zone	0 (0)	0 (0)	0 (0)		
Centrum semiovale	33 (28.0)	27 (30.7)	18 (27.3)		
Thalamus	13 (11.0)	12 (13.6)	7 (10.6)		
Brain stem	10 (8.5)	6 (6.8)	2 (3.0)		
Cerebellum	0 (0)	0 (0)	0 (0)		
Optical radiation	2 (1.7)	2 (2.3)	2 (3.0)		
**Cognitive tests scores [median (QR1–QR3)]**					
ACE-R	90 (81.5–94) (*n* = 68)	90 (81–94) (*n* = 58)	90 (81–94) (*n* = 42)	92 (84.75–96) (*n* = 63)	91 (84–96) (*n* = 65)
NART	38 (29–42) (*n* = 67)	38 (29–42) (*n* = 57)	38 (30–42) (*n* = 41)	41 (33.25–45) (*n* = 61)	32.5 (24–38.5) (*n* = 66)

*When data has been generated from a sample size different from the number of patients enrolled in each visit, this is indicated as (n = sample size). Data includes patients with more than one RSSI lesion cluster*.

* *Median of the distributions from sample that provided cognitive data differed from that from sample that did not provide cognitive data at this time point*.

#### Brain Measurements

Volumes of the index lacunar infarct and old infarcts lesions in the subsamples that provided data at the three time points were not different. The subsample that provided cognitive data at 3 years had smaller WMH median volume (*p* = 0.024, Table 1, see Supplementary Table 1 for missing values analysis). In a year-period median WMH volume increased while index stroke lesion volume decreased.

#### Cognitive Scores

The median scores for both ACE-R and NART tests followed the same pattern between the three time points: a modest increase (not statistically significant) from baseline (ACE-R = 90 [81.5–94], NART = 38 [29–42]) (median [Q1–Q3]) to the 1 year follow-up (ACE-R = 92 [84.75–96], NART = 41 [33.25–45]) followed by a decrease (also not statistically significant) in both values at the 3 year follow-up (ACE-R = 91 [84–96], NART = 32.5 [24–38.5]). Median baseline cognitive scores of the subsamples that provided data at baseline, 1 and 3 years were not different.

#### Vascular Risk Factors

Approximately 70% of the patients were hypertensive and around 60% had hyperlipidemia at baseline. These proportions did not change in the subsamples followed up at 1 and 3 years (Table 1). However, the proportion of current smokers was 39% in the baseline sample, 37.5% in the sample that provided data at 1 year and 31.8% in the sample that provided cognitive data at 3 years. The proportion of diabetics also followed similar tendency: 10.2% at baseline, 9% in the 1 year sample and 4.5% in the 3 year sample (Table 1) (see Supplementary Table 1 for missing values analysis).

### Small Subcortical Infarct Lesions—Incidence at Baseline

From the 118 patients presenting with a lacunar stroke syndrome, 79 had acute lesions (RSSI) identifiable in brain MRI. One patient had two RSSI lesion clusters in the centrum semiovale in the same cerebral hemisphere, and one patient had a bilateral RSSI in the internal/external capsule. Three patients had additional cortical DWI-positive lesions. One patient had an RSSI lesion cluster in the internal/external capsule and other in the brainstem. The rest had only one RSSI lesion cluster. Old small subcortical infarcts (lacunes) were identified in 77/118 patients. Multiple lacunes were identified in 20/118 patients. The highest frequency of RSSI and lacunes was in the internal and external capsule/lentiform nucleus and centrum semiovale (Table 1). The RSSI and lacunes were distributed fairly evenly in left and right hemispheres.

### RSSI Location and Cognitive Scores at 1 and 3 Years

**Figure 1:**
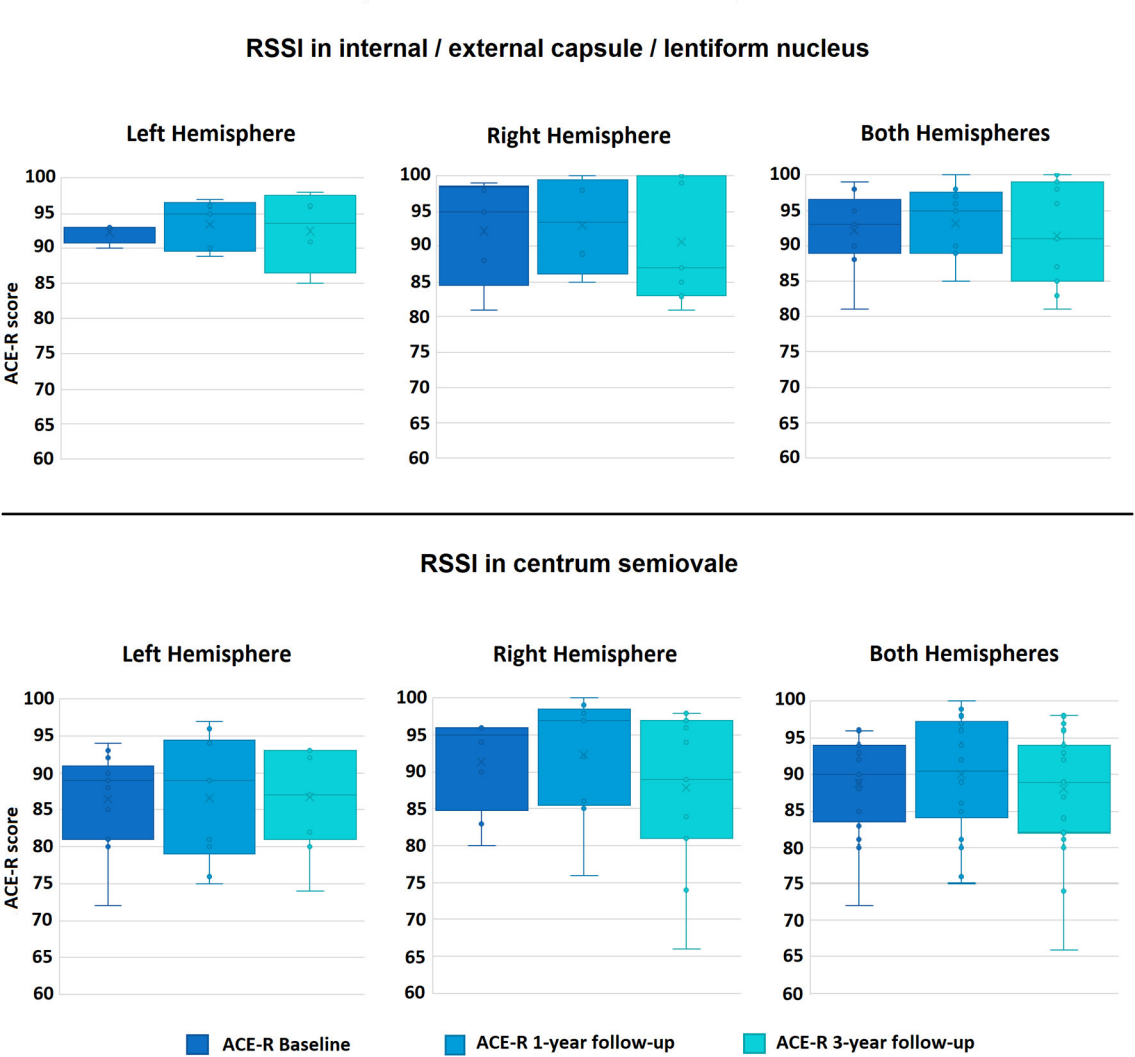
Boxplots of the distributions of the Addenbrooke's Cognitive Examination-Revised (ACE-R) scores in the sample at the three time-points, for the RSSI locations with most data: internal/external capsule/lentiform nucleus (top row), and centrum semiovale (bottom row). ACE-R scores range from 0 to 100.

Figure 1 shows the distribution of the cognitive scores at the three time points for patients with the RSSI in the two locations with more incidence in the sample. The cognitive scores across years and between patient groups were not different.

For patients with the RSSI in the internal/external capsule/lentiform nucleus (*n* = 21), baseline ACE-R scores (93 [89–96.5] (median [Q1–Q3])) increased at 1 year follow-up examination (95 [89–97.5]), but experienced an overall decrease at 3 years (91 [85–99]). Interestingly, patients with the RSSI in this location at the right cerebral hemisphere experienced, overall, a gradual decrease from baseline across the 3 year period (i.e., baseline ACE-R 95 [84.5–98.5]; 1 year ACE-R 93.5 [86–99.5] and 3 year ACE-R 87 [83–100]) (Figure 1, upper row).

Patients with the RSSI in the centrum semiovale at the right hemisphere had, overall, higher ACE-R scores at the three-time-point examinations that those who had the RSSI in the centrum semiovale at the left hemisphere (Figure 1, bottom row). Although the overall pattern of the ACE-R scores over the 3 year period resembles that of the patients with the RSSI in the internal/external capsule/lentiform nucleus, the median change in the cognitive scores from baseline to 1 year follow-up was not as pronounced (Figure 1, bottom row).

Table 2 shows the results of the ANCOVA analyses for RSSI at the internal/external capsule/lentiform nucleus and centrum semiovale, per-cerebral hemisphere and in both hemispheres combined. RSSI in these two locations did not predict total ACE-R scores at 1 or 3 years. There were insufficient patients with RSSI in other locations to conduct this analysis (see Table 1).

**Table 2:** One-way analyses of covariance (ANCOVA) results showing baseline predictors for potential change in ACE-R cognition score from baseline at 1 and 3 years follow-up for RSSI in the only two locations in which this analysis was possible due to sample size (see Table 1): internal/external capsule/lentiform nucleus (i.e., abbreviated as BG as per basal ganglia) and centrum semiovale (i.e., abbreviated as CSO).

**Outcome—dependent variable**	**Predictor—independent variable**	**Covariates [B (SE)]**										
		**Lesion location**		**Diabetes**	**Hypertension**	**Hyperlipidemia**	**Smoking**	**Sex**	**Age**	**WMH**	**NART score at baseline**	**ACE-R baseline predictor**
**Predictors of cognitive performance at 1 and 3 years follow up from baseline**												
ACE-R score at 1 year follow up	ACER-R baseline score	BG RH	3.407 (4.458)	2.334 (4.705)	1.092 (2.297)	−0.550 (4.234)	0.239 (1.483)	−0.744 (1.258)	−0.127 (0.168)	0.633 (0.675)	0.255 (0.1954)	89.849 (14.540) (*p* = 0.000)
	ACER-R baseline score	BG LH	−3.331 (6.394)	3.507 (5.211)	1.059 (5.005)	−0.297 (3.777)	−0.021 (1.552)	−0.486 (2.012)	−0.106 (0.155)	0.427 (0.713)	0.222 (0.296)	87.460 (17.858) (*p* = 0.000)
	ACER-R baseline score	BG combined	−0.303 (5.005)	3.909 (4.992)	0.715 (2.510)	−0.238 (4.576)	−0.025 (1.404)	−0.748 (2.237)	−0.100 (0.131)	0.464 (0.716)	0.230 (5.005)	86.816 (14.934) (*p* = 0.000)
	ACER-R baseline score	CSO RH	−5.574 (3.591)	4.080 (4.768)	−0.163 (1.903)	0.255 (3.409)	−0.096 (0.965)	−0.767 (1.904)	−0.091 (0.118)	0.448 (0.763)	0.245 (0.201)	86.127 (17.303) (*p* = 0.000)
	ACER-R baseline score	CSO LH	−1.615 (9.934)	3.598 (4.631)	0.674 (5.962)	0.024 (3.336)	0.095 (2.137)	−0.985 (2.782)	−0.085 (0.230)	0.553 (0.955)	0.232 (0.350)	85.975 (17.486) (*p* = 0.000)
	ACER-R baseline score	CSO combined	−3.249 (3.882)	3.614 (7.844)	−0.004 (2.810)	0.662 (4.172)	0.133 (1.739)	−1.238 (2.519)	−0.060 (0.164)	0.609 (0.577)	0.246 (0.260)	84.253 (26.093) (*p* = 0.00)
ACE-R score at 3 year follow-up	ACER-R baseline score	BG RH	−2.666 (0.989)	2.577 (1.557)	−1.414 (3.090)	2.535 (0.544)	−0.151 (0.087)	0.295 (0.091)	−0.163 (0.289)	−0.928 (0.428)	0.312 (0.037)	91.643 (17.691) (*p* = 0.001)
	ACER-R baseline score	BG LH	−1.378 (9.801)	0.223 (6.650)	−1.095 (4.437)	2.555 (4.499)	−0.049 (10.331)	0.640 (7.715)	−0.166 (0.133)	−0.890 (1.116)	0.284 (1.078)	96.024 (39.600) (*p* = 0.000)
	ACER-R baseline score	BG combined	−2.666 (7.808)	2.577 (2.805)	−1.414 (10.993)	2.535 (10.334)	−0.151 (10.373)	0.295 (9.929)	−0.163 (0.425)	−0.928 (0.638)	0.312 (1.150) (*p* = 0.069)	91.643 (26.599) (*p* = 0.001)
	ACER-R baseline score	CSO RH	−1.079 (2.815)	0.372 (4.962)	−1.084 (4.463)	2.285 (0.804)	−0.058 (1.0490)	0.609 (6.116)	−0.170 (0.177)	−0.843 (0.882)	0.291 (0.448)	95.633 (20.434) (*p* = 0.000)
	ACER-R baseline score	CSO LH	1.082 (36.163)	0.002 (104.539)	−0.899 (83.621)	2.376 (92.679)	−0.153 (68.670)	0.715 (169.394)	−0.175 (5.379)	−0.926 (10.075)	0.281 (14.837)	96.947 (1337.516) (*p* = 0.000)
	ACER-R baseline score	CSO combined	1.082 (143.507)	0.002 (98.533)	−0.899 (36.009)	2.376 (32.975)	−0.153 (59.433)	0.715 (87.776)	−0.175 (2.725)	−0.926 (12.739)	0.281 (3.868)	96.947 (768.868) (*p* = 0.000)

*L and R refer to the left and right hemisphere. The association (B; i.e., non-standardized B coefficient) and standard error (SE) from all terms of the models are given. p-values are only given if significant (p < 0.05)*.

### Voxel-Wise Association of 1 Year WMH Change and Cognitive Scores at 1 and 3 Years

#### 1 Year WMH Change

**Figure 2:**
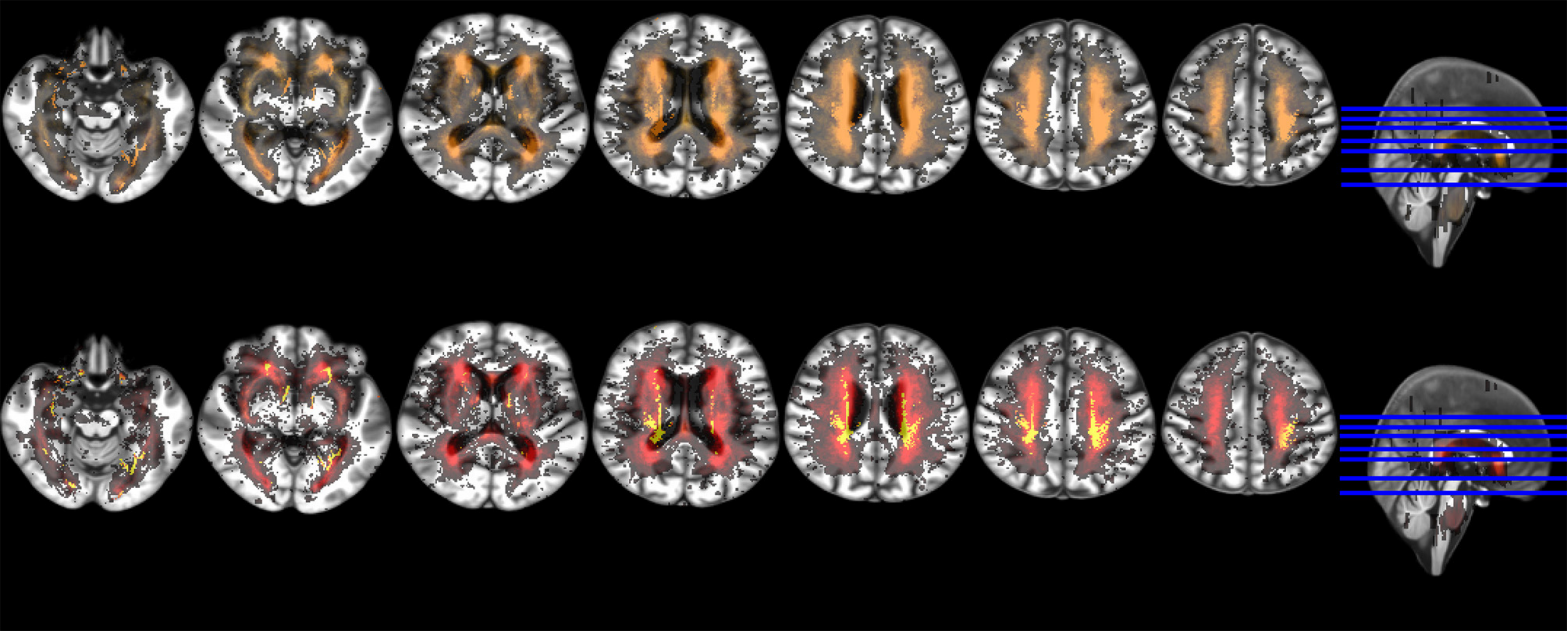
Axial slices of probability distribution maps of WMH change from baseline to 1 year overlaid on the brain study template. The upper row shows the baseline probability distribution of WMH in red with probability distribution of WMH change in yellow superimposed at 50% transparency. The bottom row shows the probability distribution of WMH change in red with the probability distribution of only the WMH that disappeared at follow-up added in yellow.

Change in WMH in the sample was widespread across a large number of voxels (Figure 2). WMH disappeared mainly in localized clusters of the centrum semiovale and around the ventricles. These regions correspond to those with prevalence of old lacunes and ventricular enlargement (Figure 2, bottom row).

#### Association Between WMH Change and Cognitive Scores at 1 Year

**Figure 3:**
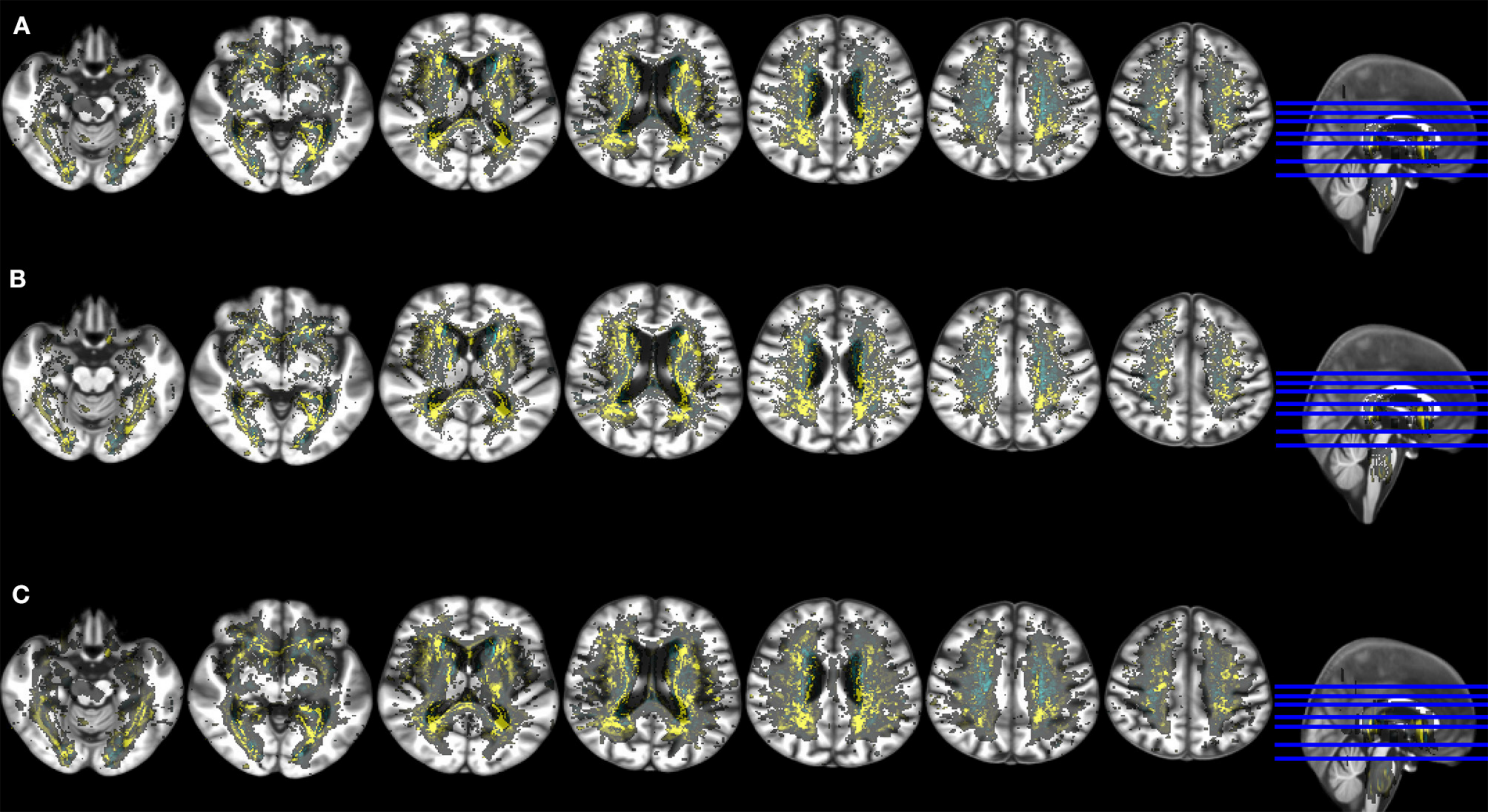
Axial slices of the study brain template with overlays showing the voxel-wise association (non-standardized B-values) between ACE-R at 1 year (outcome variable) and spatial distribution of WMH change (main predictor) in models that accounted for age, premorbid cognition and: **(A)** baseline WMH volume, number of lacunes, RSSI location and vascular risk factors (upper row), **(B)** baseline WMH volume, number of lacunes and RSSI location (middle row), **(C)** baseline WMH volume and vascular risk factors (bottom row). Negative B-values are shown in yellow and positive B-values (no WMH change or WMH disappearance → reduction of ACE-R scores at 1 year) in cyan. These results were obtained using in the model the RSSI location of the primary DWI positive cluster.

Figure 3 shows the non-standardized voxel-based associations (B-values) between WMH change and ACE-R scores 1 year post-stroke from different models. While vascular risk factors practically did not influence these associations (upper vs. middle rows in Figure 3, Table 3), RSSI location did (Figure 3, bottom row vs. the upper and middle rows). Not accounting for RSSI location or lacunes in the model (Figure 3, bottom row) slightly increased the strength (Table 3) and extent of the associations and influenced the pattern of their spatial distribution.

**Table 3:** Median, interquartile range (IQR) and extreme B values of the voxel-wise effect of WMH change in cognition at 1 and 3 years post-stroke.

**Outcome variable**	**Model covariates (in addition to baseline NART and age)**	**Positive B-values median (QR1 QR3) (×10^**−3**^)**	**Positive B-values [min max] (×10^**−3**^)**	**Negative B-values median (QR1 QR3) (×10^**−3**^)**	**Negative B-values [min max] (×10^**−3**^)**
ACE-R 1 year	RSSI location	0.118 (0.028 0.343)	[0 8.087]	−0.14 (−0.4 −0.030)	[−7.13 0]
	RSSI location and VRF	0.119 (0.030 0.345)	[0 8.075]	−0.14 (−0.41 −0.033)	[−7.14 0]
	Baseline WMH vol	0.119 (0.030 0.344)	[0 8.117]	−0.13 (−0.40 −0.030)	[−7.12 0]
	Baseline WMH vol and VRF	0.118 (0.029 0.342)	[0 8.148]	−0.13 (−0.40 −0.029)	[−7.11 0]
	Baseline WMH vol, number of lacunes, and RSSI location	0.118 (0.029 0.344)	[0 8.084]	−0.14 (−0.41 −0.032)	[−7.13 0]
	Baseline WMH vol, number of lacunes, RSSI location and VRF	0.119 (0.030 0.346)	[0 8.100]	−0.14 (−0.41 −0.033)	[−7.11 0]
	Baseline WMH vol, number of lacunes, old stroke lesion volume and RSSI location	0.116 (0.027 0.340)	[0 8.089]	−0.14 (−0.40 −0.030)	[−7.12 0]
	Baseline WMH vol, number of lacunes, old stroke lesion volume, RSSI location and VRF	0.117 (0.028 0.342)	[0 8.096]	−0.14 (−0.41 −0.031)	[−7.12 0]
ACE–R 3 years	RSSI location	0.132 (0.037 0.372)	[0 5.695]	−0.14 (−0.40 −0.042	[−6.53 0]
	RSSI location and Vascular risk factors	0.132 (0.036 0.371)	[0 5.685]	−0.14 (−0.40 −0.041)	[−6.49 0]
	Baseline WMH vol	0.132 (0.037 0.371)	[0 5.674]	−0.14 (−0.40 −0.041	[−6.50 0]
	Baseline WMH vol and VRF	0.131 (0.036 0.371)	[0 5.658]	−0.14 (−0.40 −0.041)	[−6.51 0]
	Baseline WMH vol, number of lacunes, and RSSI location	0.135 (0.040 0.377)	[0 5.670]	−0.15 (−0.41 −0.045	[−6.50 0]
	Baseline WMH vol, number of lacunes, RSSI location and VRF	0.134 (0.039 0.378)	[0 5.692]	−0.15 (−0.41 −0.045)	[−6.50 0]
	Baseline WMH vol, number of lacunes, old stroke lesion volume and RSSI location	0.132 (0.037 0.371)	[0 5.678]	−0.14 (−0.40 −0.041	[−6.51 0]
	Baseline WMH vol, number of lacunes, old stroke lesion volume, RSSI location and VRF	0.131 (0.036 0.370)	[0 5.670]	−0.14 (−0.40 −0.041)	[-6.52 0]

*VRF, vascular risk factors; RSSI, recent small subcortical infarct. Only voxels with non-zero B-values were considered for calculating the median and interquartile range (IQR) values in this table. The RSSI location includes all DWI-positive RSSI lesion clusters. Excluding secondary clusters yielded results only differing in the order of 10^−6^*.

#### Association Between 1 Year WMH Change and Cognitive Scores at 3 Years

**Figure 4:**
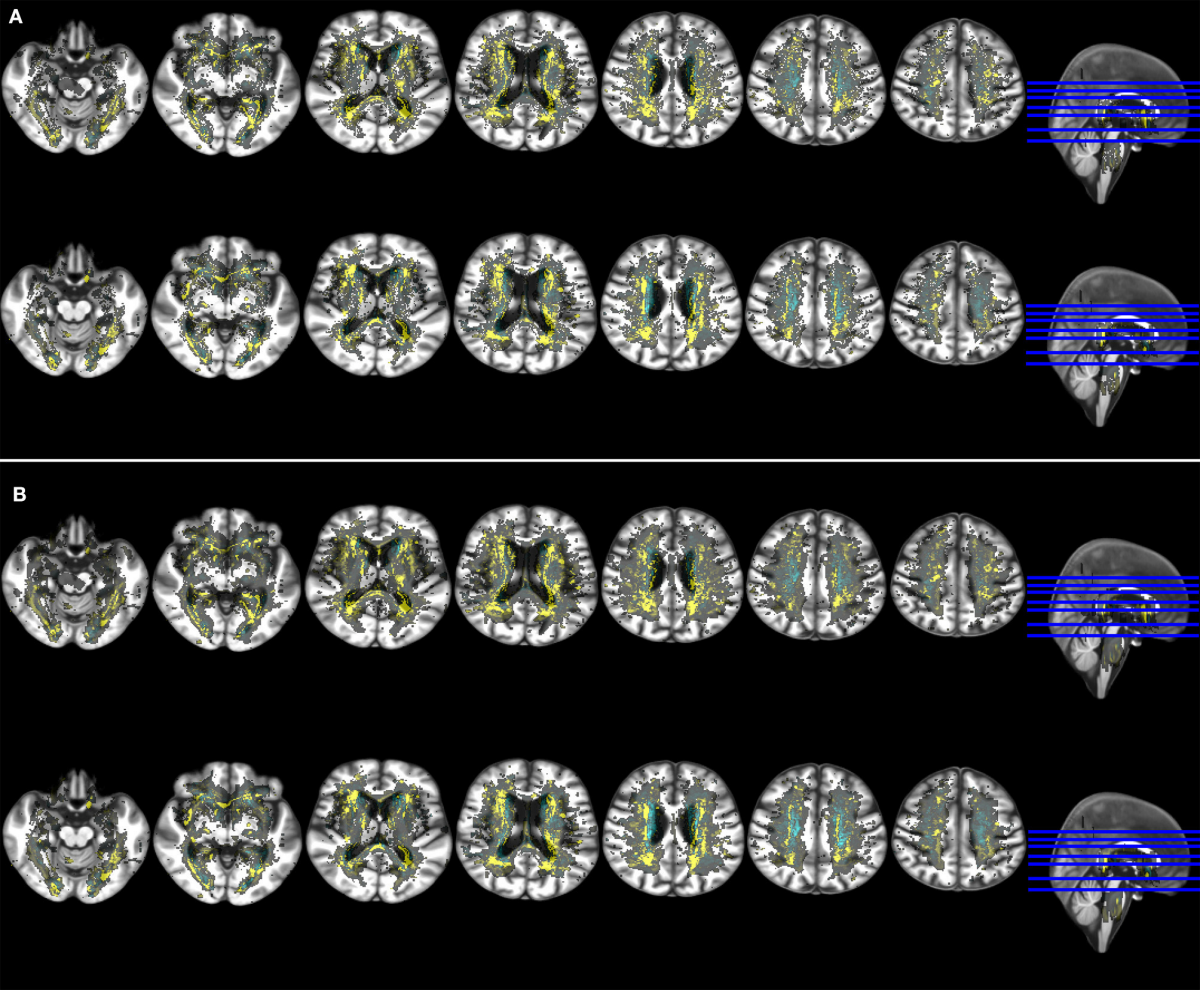
Axial slices of the study brain template with overlays showing the voxel-wise association (non-standardized B-values) of ACE-R (outcome variable) at 1 year (upper row in both panels) and 3 year post stroke (bottom row in both panels) with spatial distribution of WMH change (main predictor). Models accounted for age, premorbid cognition and: **(A)** baseline WMH volume, number of lacunes, RSSI location and vascular risk factors (upper panel), **(B)** baseline WMH volume and vascular risk factors (bottom panel). Negative B-values are shown in yellow and positive B-values (no WMH change or WMH disappearance → reduction of ACE-R scores at 1 year) in cyan. These results were obtained using in the model the RSSI location of the primary DWI positive cluster.

Regardless of considering or not the RSSI location as covariate in the models, the strength of the associations between WMH change and cognition generally increased at 3 years with respect to 1 year (see Figure 4, Table 3), especially in voxels with no change or disappearance of WMH at 1 year, the latter mainly due to tissue loss (voxels in cyan in Figure 4), in the splenium of the corpus callosum and at the intersection between the anterior limb of the internal and external capsules. Only in the external capsule and occipito-parietal locations contiguous to the lateral ventricles, the associations were stronger at 1 year than 3 years after the stroke. To add the volume of old stroke lesions as covariate in the models weakened the median strength of the voxel-based associations at 1 year but did not make any difference at 3 years (Table 3).

RSSI location did not influence the associations between WMH change and cognition at 3 years as markedly as at 1 year post-stroke, except in the region of the lentiform nucleus (Figure 4). In models that only accounted for RSSI location, age and pre-morbid cognition, clusters of WMH change negatively and more strongly associated with ACE-R at 1 and 3 years were mainly in the external capsule, intersection between the anterior limb of the internal and external capsules, and optical radiation (Supplementary Figures 1, 2).

## Discussion

### Main Findings

In our sample of patients who presented to the clinic with a mild stroke syndrome of type lacunar, the location of the acute lesion in the two locations with more incidence: internal/external capsule/lentiform nucleus and centrum semiovale, did not predict the general cognitive outcome at 1 or 3 years after. However, lacunar stroke lesion location moderated the association between WMH evolution (i.e., appearance and disappearance) 1 year post-stroke, and cognition at 1 and 3 years.

This underlines previous evidence that post-stroke cognitive impairment in lacunar stroke is, in general, unrelated to the location of the lesion, but instead related to the extent of the underlying small vessel disease pathology which is often diffuse throughout the subcortical tissues (41). This is not surprising, given that lacunar stroke, by definition, does not cause higher cortical dysfunction. Clinicians treating patients with lacunar stroke should consider all participants to be at risk of cognitive impairment.

Despite the acute lesions being located mainly in the centrum semiovale, internal/external capsule/lentiform nucleus in our sample, we can't assert whether a larger balanced sample in terms of RSSI locations would yield different results or not. In a sample of 182 patients with probable vascular dementia presenting infratentorial vascular abnormalities (i.e., 65.4% had focal infratentorial vascular lesions and 35.7% had diffuse pontine vascular abnormalities hyperintense on T2-weighted images), these were not associated with cognition or cognitive decline (42), but much larger samples are needed to confirm these findings.

The results of our voxel-based analysis WMH are in agreement with a previous study of patients with acute ischemic lesions in the left cerebral hemisphere, which concluded that the corpus callosum, corona radiata, and posterior thalamic radiation could be strategic substrates for short-term and long-term post-stroke cognitive impairment (15). In our sample of lacunar stroke patients we also found that some WMH clusters strongly associated with cognition 1 year post-stroke differ from some others that are rather more strongly associated with the cognitive performance 3 years after the stroke, perhaps reflecting a complex dynamic in WMH evolution influencing different cognitive functions.

### Secondary Findings

Patients with the RSSI in the centrum semiovale in the right hemisphere had greater increase in ACE-R scores at 1 year than those with the RSSI at the left hemisphere, in agreement with previous study on cognitive recovery 2 years post-stroke (43). However, this hemispheral difference did not reach statistical significance and did not hold for the longer term cognitive outcome (i.e., 3 years), which was poorer than baseline in general. The apparent cognitive improvement at 1 year post-stroke has been reported before. A study of silent brain infarcts in 1,015 individuals aged 60–90 years old, found an increase in the cognitive performance at the second examination (equivalent to our 1 year follow up) (44). Other two studies including the sample analyzed here and a similar number of patients with mild-to moderate cortical strokes found similar trends overall: a slightly better cognitive performance at 1 year and an overall cognitive decline at 3 years regardless of stroke subtype (36, 40), in agreement with previous investigations in the stroke literature (45). Declining cognition is a characteristic of aging and presents a challenge to our study as with a population average of 65 (at baseline) it could be difficult to separate cognitive decline due to lacunar stroke than the decline caused by aging despite all our models having accounted for age. Where possible studying a younger population of stroke patients would enable the separation of age-related cognitive decline.

### Strengths and Limitations

The uniformity in assessing the population in terms of MRI acquisition and processing protocols, the application of well-established guidelines, the use of state-of-the-art imaging processing methods and the detailed clinical and neuroradiological assessments are strengths of this study. The machine-learning voxel-based approach used, made publicly available, is also a strength. The sensitivity analysis considering the RSSI location of (a) only the main DWI-positive RSSI clusters and (b) all, including secondary, DWI-positive lesion clusters, confirming the overall conclusions of the study is another strength. Although moderate in size, our sample was affected by the reduction in the number of patients attending follow-up assessments and missing cognitive data in all time points (see Table 1). Analyses of RSSI location per brain hemisphere and cognition in specific domains were limited due to reduced sample-size at the follow-up examinations and skeweness of the data. We, therefore, could not include all predictors of potential interest (e.g., brain microbleeds) to avoid overfitting in our models. Moreover, patients who provided cognitive data at 3 years were healthier at baseline (i.e., higher proportion of no smokers and ex-smokers, and less WMH volume). It is not uncommon that subsamples are healthier with length of follow-up. Although it seems reasonable to think that patients who dropped out had greater cognitive decline, they could not be included in the analysis. Another limitation to the study was the lack of control group for direct comparison between age-matched individuals, which would have provided a better picture of the influence of the acute lacunar stroke in the cognitive changes at 1 and 3 years after the baseline examination.

### Future Directions

Future research should aim to increase the follow-up period of the study beyond the 3 year period studied here. In addition, efforts should be made to increase the sample size of lacunar stroke patients, to increase the reliability and power of the statistical analysis. Creating an international database could provide a promising solution to the small sample sizes which most of the recent literature on lacunar or other mild cortical strokes lists as a limitation, initiated either as a new project or as a branch of a project/consortia like the PLORAS database (46) or the Meta VCI Map consortium (17). A range of cognitive tests has been used by different studies making comparison between studies challenging. There is evidence of the combined benefits of using a domain-specific cognitive test (e.g., ACE-R) and premorbid intelligence test (e.g., NART) (38). Unified recommendations are needed for accurate estimates in the study of cognition after stroke. The development of a digital resource that could facilitate patient cognitive, focal and non-focal symptoms screening at home feeding to a secure data heaven for follow-up and research purposes will be also beneficial. This could be particularly useful when looking to study cognitive performance over several years and would enable better prediction of cognitive changes at different times over a longer follow-up period.

## Data Availability Statement

The original contributions presented in the study are included in the article/Supplementary Material, further inquiries can be directed to the corresponding author/s.

## Ethics Statement

The studies involving human participants were reviewed and approved by The Lothian Ethics of Medical Research Committee (REC 09/81101/54) and NHS Lothian R+D Office (2009/W/NEU/14), on the 29th of October 2009. The patients/participants provided their written informed consent to participate in this study.

## Author Contributions

MV: image processing and analysis, data analysis, study design, and writing and approving the manuscript. TG-M: data analysis and writing and approving the manuscript. FC: statistical analysis, editing, and revising and approving the manuscript. MT and PA: image protocol design, image data quality control, editing, and revising and approving the manuscript. SM: patient recruitment, data generation, application of cognitive tests, clinical examination, editing, and revising and approving the manuscript. JW: study design, project supervision, funding, editing, and revising and approving the manuscript. All authors contributed to the article and approved the submitted version.

## Conflict of Interest

The authors declare that the research was conducted in the absence of any commercial or financial relationships that could be construed as a potential conflict of interest.
