# Supplementary figures and images for: Post-stroke Cognition at 1 and 3 Years Is Influenced by the Location of White Matter Hyperintensities in Patients With Lacunar Stroke
Source: Front Neurol. 2021 Mar 1;12:634460. doi: 10.3389/fneur.2021.634460 (PMC7956970; doi:10.3389/fneur.2021.634460)

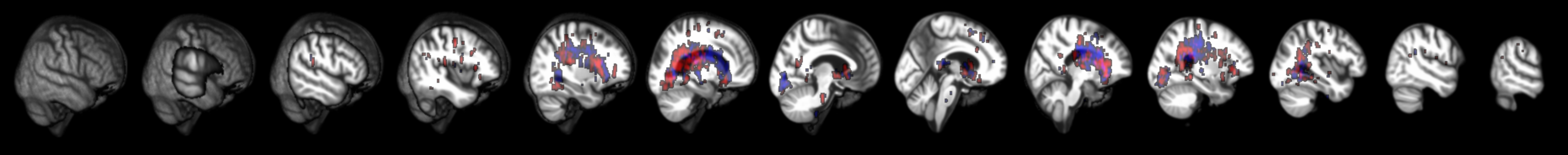

Supplement: Supplementary file 1 [file Image_1.TIFF]

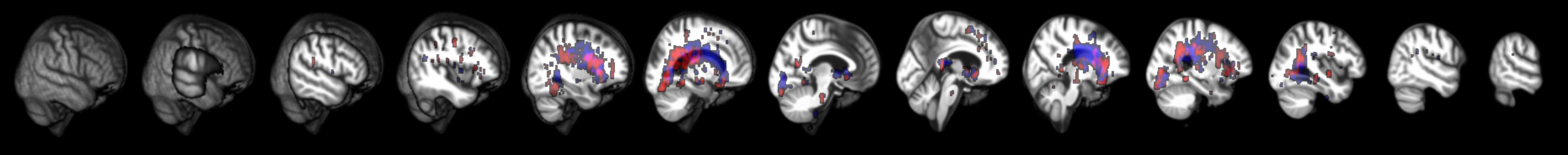

Supplement: Supplementary file 2 [file Image_2.TIFF]
