# Supplementary material for: Post-stroke Cognition at 1 and 3 Years Is Influenced by the Location of White Matter Hyperintensities in Patients With Lacunar Stroke
Source: Front Neurol. 2021 Mar 1;12:634460. doi: 10.3389/fneur.2021.634460 (PMC7956970; doi:10.3389/fneur.2021.634460)
Supplement: Supplementary file 3 [file Table_1.DOCX]

**Post-stroke cognition at 1 and 3 years is influenced by the location of white matter hyperintensities in patients with lacunar stroke**

Maria del C. Valdés Hernández, Tara Grimsley-Moore, Francesca M. Chappell, Michael J. Thrippleton, Paul A. Armitage, Eleni Sakka, Stephen Makin and Joanna M. Wardlaw

Supplementary Table 1.

Missing values analysis - Results of the Mann-Whitney U test of different parameters comparing the subsamples that had cognitive tests at each time point vs. the subsamples that did not provide cognitive tests results at the time points specified.

| Parameter tested | p-value | h-value | Wilcoxon rank sum statistic |
| --- | --- | --- | --- |
| Age at baseline | 0.388 | 0 | 3134 |
| Age at 1 year | 0.463 | 0 | 3409 |
| Age at 3 years | 0.203 | 0 | 3389.5 |
| Diabetes at baseline | 0.242 | 0 | 3088 |
| Diabetes at 1 year | 0.0388 | 1 | 3473.5 |
| Diabetes at 3 years | 0.0281 | 1 | 3366.5 |
| Hypertension at baseline | 0.766 | 0 | 2931 |
| Hypertension at 1 year | 0.377 | 0 | 3141.5 |
| Hypertension at 3 years | 0.259 | 0 | 2985.5 |
| Hyperlipidaemia at baseline | 0.134 | 0 | 2743 |
| Hyperlipidaemia at 1 year | 0.129 | 0 | 3035 |
| Hyperlipidaemia at 3 years | 0.938 | 0 | 3166 |
| Smoker status at baseline | 0.059 | 0 | 3214 |
| Smoker status at 1 year | 0.010 | 1 | 3629.5 |
| Smoker status at 3 years | 0.111 | 0 | 3402.5 |
| WMH volume at baseline | 0.359 | 0 | 3144 |
| WMH volume at 1 year | 0.539 | 0 | 3387 |
| WMH volume at 3 years | 0.024 | 1 | 3572 |
| RSSI volume at baseline | 0.640 | 0 | 2890.5 |
| RSSI volume at 1 year | 0.340 | 0 | 3099 |
| RSSI volume at 3 years | 0.982 | 0 | 3158 |
| Old strokes lesion volume at baseline | 0.490 | 0 | 2866 |
| Old strokes lesion volume at 1 year | 0.845 | 0 | 3241 |
| Old strokes lesion volume at 3 years | 0.515 | 0 | 3257 |

The p-value of the test (0≤p≤1), is the probability of observing the test statistic as or more extreme than the observed value under the null hypothesis. The MATLAB function used computes the two-sided p-value by doubling the most significant one-sided value.

The h-value is the result of the hypothesis test returned as a logical value.

If h = 1, the null hypothesis that the medians of the distributions of the two subsamples are equal is rejected at the 100 * alpha% significance level.

If h = 0, the null hypothesis could not be rejected, also at the 100 * alpha% significance level.
